# Use of Gabapentin or Alprazolam in Cats during Postoperative, Short-Term Hospitalization

**DOI:** 10.3390/ani14131840

**Published:** 2024-06-21

**Authors:** Virginia Papageorgiou, Charalampos Ververidis, Mathios E. Mylonakis, Ioannis Savvas, George Kazakos

**Affiliations:** Companion Animal Clinic, School of Veterinary Medicine, Aristotle University of Thessaloniki, 11 Stavrou Voutyra St., 54627 Thessaloniki, Greece; harisver@vet.auth.gr (C.V.); mmylonak@vet.auth.gr (M.E.M.); isavas@vet.auth.gr (I.S.); gkdvm@vet.auth.gr (G.K.)

**Keywords:** feline, alprazolam, gabapentin, hospitalization, stress

## Abstract

**Simple Summary:**

Stress during hospitalization can significantly impact cats, prompting the exploration of various strategies, including non-pharmacological approaches, to alleviate stress. However, certain situations may necessitate the use of anxiolytic medications like gabapentin and alprazolam to reduce stress levels. This study aimed to assess the effectiveness of gabapentin or alprazolam in cats undergoing short-term hospitalization on stress reduction, food consumption, and pain scores. Sixty cats undergoing elective ovariohysterectomy were divided into three groups, gabapentin-, alprazolam- or placebo-treated, and received treatment twice daily for two days. The study found that both gabapentin and alprazolam significantly reduced stress levels. Cats treated with alprazolam exhibited increased food intake, while those receiving gabapentin showed lower pain scores on the first postoperative day. However, both treatments led to increased levels of sedation. The study suggests that incorporating anxiolytics may be an effective adjunctive strategy for improving the experience of short-term hospitalization in cats.

**Abstract:**

This prospective, randomized study aimed to assess the anxiolytic efficacy of gabapentin or alprazolam in cats during short-term postoperative hospitalization. Sixty cats were randomly assigned to three groups (gabapentin-treated [100 mg per cat], alprazolam-treated [0.125 mg per cat], or placebo-treated), with treatments administered twice daily for two days. Stress levels were evaluated using Cat Stress Scores, serum cortisol, and glucose concentrations. Pain scores, food consumption, and adverse effects such as sedation were also monitored. Fifty-five cats completed the study. Both medications demonstrated similar reductions in stress levels. Cats receiving gabapentin had lower pain scores, while those receiving alprazolam exhibited significantly increased food intake on the first postoperative day. However, both medications resulted in comparable levels of sedation. In the context of postoperative hospitalization, pharmacological intervention with anxiolytics could be effective in reducing stress levels. Despite potential side effects, gabapentin and alprazolam may contribute to an improved quality of short-term hospitalization for cats.

## 1. Introduction

Hospitalization is often an unpleasant experience for animals, as it is associated with loss of environmental control and exposure to potentially painful or stressful situations [1]. In veterinary practice, hospitalization is related to the separation of the animal from its owner and its confinement in a cage that triggers fear and anxiety. The animal is exposed to numerous stressors including unfamiliar sounds, smells, people, and painful stimuli, which dramatically upset its routine [2,3]. Stressors can be physiological, arising from disease or trauma, or psychological. The resultant stress is a natural response helping the animal to cope with disruptions of homeostasis, but it can also lead to various adverse effects [4].

In shelter cats, increased stress levels have been correlated with reduced food intake and weight loss, even if palatable food is available [5]. Although hospitalized cats have overall low energy needs, they may enter a catabolic state due to disease or surgical trauma. In this setting, proteolysis and high energy requirements may cause loss of lean body mass [6]. Reluctance to consume food may also delay surgical wound healing and prolong the recovery period [7]. Therefore, early postoperative food consumption is crucial for providing adequate nutritional support [2].

Tachycardia, hypertension, hyperthermia, and hyperventilation are common findings during physical examination of stressed cats [3]. Neutrophilia, lymphopenia, hypokalemia, and hyperglycemia, with or without glycosuria, may also be observed [3,8]. In addition, stressed cats may present increased scores in pain scales [9] and may require more postoperative analgesic drugs to achieve adequate analgesia [4]. Moreover, a cat frightened during hospitalization might attempt to escape from its stressful environment (flight response) or exhibit aggression towards the personnel (fight response) [2,10].

Various methods have been suggested to minimize stress in hospitalized cats. These include environmental alternations in the hospitalization areas, such as providing a hiding place inside the cage [11], implementing music therapy [12], and using pheromones [4]. Furthermore, techniques such as placing a towel or using plexiglass in the front of the cage to attenuate visual and auditory stressors have been utilized [13,14]. Recently, the use of anxiolytic medications has been suggested to minimize stress [2]. To date, few anxiolytic drugs have been used in cats to manage pre-appointment stress [15] and only one study has evaluated the use of daily gabapentin in shelter cats to reduce stress and improve behavior [16]. To the best of the authors’ knowledge, no studies have assessed the use of gabapentin or alprazolam as anxiolytic medications in cats during hospitalization. The aim of our study was to assess the anxiolytic effects of gabapentin or alprazolam in cats during short-term postoperative hospitalization. We hypothesized that both of these medications would result in reduced stress and increased food intake compared to placebo.

## 2. Materials and Methods

### 2.1. Animals

This manuscript is a continuum of a recently published study, which assessed the anxiolytic and sedative effects of a single administration of alprazolam or gabapentin in cats in a preoperative setting [17]. Briefly, client-owned cats admitted to the Companion Animal Clinic, School of Veterinary Medicine, Aristotle University of Thessaloniki (CAC-AUTh), Greece, for elective ovariohysterectomy were enrolled in the study. Owners provided informed written consent before the inclusion of the cats in the study. This prospective, blinded, controlled clinical trial received ethical approval from the Ethics Committee of the School of Veterinary Medicine, AUTh (757/23-5-2022). Eligible cats were required to be at least 6 months old, weigh more than 3 kg, and have an American Society of Anesthesiologists physical status of 1. A dry-food-only diet was another inclusion criterion for the cats.

**Figure 1:**
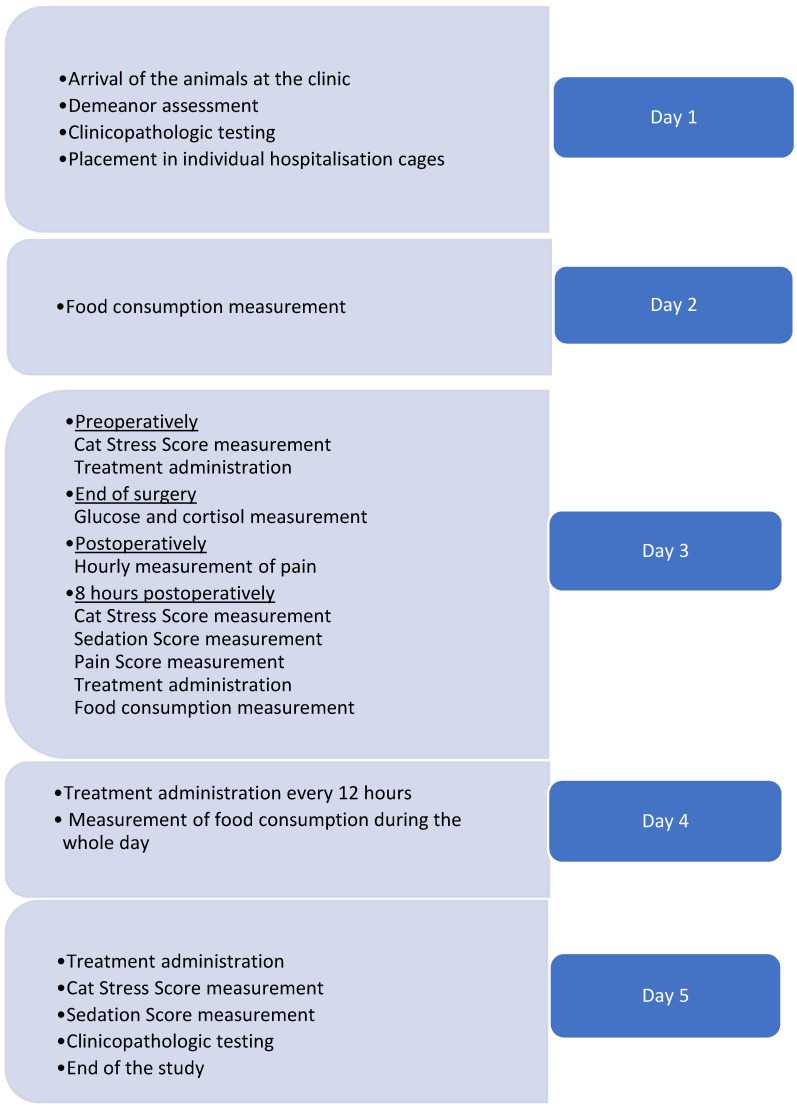
Timetable of the major events from the admission of cats to the clinic to the end of the experimental study (5 days).

Upon admission to the clinic (Figure 1), cats were transported to a quiet room where they remained for approximately an hour. Afterwards, the primary investigator (VP) assessed their demeanor using the “Scale of handling” [18], which ranged from 1 (easy to handle) to 5 (extremely aggressive). Animals with a score of 5 were excluded from the study. Cats were gently restrained with a towel and a blood sample was collected for complete blood count (CBC), serum biochemistry, and serum basal cortisol measurement (Siemens Healthcare Diagnostics, Tarrytown, NY, USA), and in-house serology for the detection of feline immunodeficiency virus antibodies and feline leukemia virus antigens (FIV/FeLV Combo test, Idexx Laboratory Inc., Syracuse, NY, USA). Cats with clinically relevant clinicopathologic abnormalities or seropositivity to either of the retroviruses were excluded from the study. Cats were allocated to each group (as described below) via a randomization program (www.randomization.com; accessed on 1 May 2023). All assessments were blindly conducted by the same investigator (VP).

### 2.2. Procedure

#### 2.2.1. Preoperative Period

Animals were transferred to their hospitalization cages in a dedicated dimly lit and minimally noisy feline ward, with no visual contact between cats. Each cage was equipped with water and food utensils, a litter tray, and a cardboard box with a blanket-covered floor to provide a hiding place. The cage floor was covered with individual blankets. In addition, synthetic pheromones (Feliway, CEVA animal health, High Wycombe, UK) were utilized to reduce stress levels, and each cat was provided with an individual soft ball as an entertainment toy [17].

Cats were hospitalized for two days preoperatively to familiarize with the environment and the examiner [19]. A creamy treat (Trixie pet supplies, Tarp, Germany) was provided daily to all cats that voluntarily consumed it. During this period, the principal investigator was their sole human contact, responsible for cleaning their cages and litter trays, as well as providing them with food and water twice daily. The daily amount of dry food provided to cats matched their resting energy requirements (RER = 70 × BW kg^0.75^) [6]. Food consumption was measured on post-admission day 2, using a calibrated scale. The investigator measured the quantity of the provided dry food before and 2 h after every meal, calculating the amount of food consumed per meal.

On the third day post-admission, food was withheld and the Cat Stress Score was used to assess the cats’ stress levels [20]. Subsequently, all cats received their treatment medication, as follows: a reformulated 100 mg gabapentin capsule (Neurontin, UPJOHN, Athens, Hellas), which was emptied and admixed with the creamy treat (group G), a 0.125 mg tablet of alprazolam (Xanax, Pfizer, NY, USA) crushed and admixed with the same amount of treat (group A), or the placebo treatment which included the creamy treat with no medication (group P) [17]. Cats were excluded from the study if they did not eat all the offered treatment. Ninety minutes after treatment administration, general anesthesia was provided and cats underwent elective ovariohysterectomy, performed by the same experienced surgeon (CV). Cats were premedicated with acepromazine (Acepromazine, Alfasan, Utrecht, The Netherlands) (0.02 mg kg^−1^) and buprenorphine (Bupredine, Northwich, UK) (0.02 mg kg^−1^). Induction of anesthesia was achieved with propofol (1 mg kg^−1^ with additional doses of 1 mg kg^−1^) until orotracheal intubation could be performed. Anesthesia was maintained with isoflurane in O_2_. Meloxicam (Loxicom, Norbrook, Monaghan, Ireland) (0.1 mg kg^−1^) was administered preoperatively in all cats for additional analgesia. Furthermore, Lactated Ringer’s solution (Baxter Hellas, Athens, Greece) was administered perioperatively at a rate of 3 mL kg^−1^ h^−1^. At the end of the surgical procedure, immediately after extubation, a second blood sample was collected for cortisol and glucose measurement. Following surgery, cats were transferred back to their cages in the hospitalization area.

#### 2.2.2. Postoperative Period

Postoperatively, pain assessment was performed hourly, for 8 h, using the Glasgow Feline Composite Measure Pain Scale [21]. Cats with a pain score above 5/20 were administered tramadol (3 mg kg^−1^) for additional analgesia and were excluded from the study. Eight hours postoperatively, a clinical examination was performed and stress and pain scores were recorded. Sedation was also recorded using the Volpato Sedation Score [22]. Similarly to the preoperative period, the group-assigned treatment was administered again. Afterwards, the same dry food was offered and quantified. Treatment administration and the feeding routine were continued every 12 h until the morning of post-admission day 5 (first postoperative day). At that point, cats received only the creamy treat with their allocated medication and no food. Ninety minutes after treatment consumption, a third blood sample was obtained for CBC, serum biochemistry, serum cortisol, and glucose measurement. In addition, pain and sedation scores were assessed.

During the postoperative period (post-admission days 4 and 5), all cats received meloxicam orally (0.1 mg kg^−1^), once daily, and oral transmucosal buprenorphine (0.02 mg kg^−1^), twice daily. Furthermore, tramadol (3 mg kg^−1^) was administered if additional analgesia was required. However, these cats were then excluded from the study.

After the conclusion of the experiment, all cats were fed and remained hospitalized for one more day until they were finally discharged, 68 h postoperatively.

### 2.3. Statistical Analysis

A power analysis was performed in order to estimate the number of cats required to reduce the stress score by 2, with a 1-β error probability of 0.8 and α error probability of 0.05. A sample size of 60 animals, divided into 20 cats in each group, was required. Stress, sedation, and pain scores, as well as cortisol and glucose concentrations and food consumption, were analyzed using a General Linear Model for repeated measurements, with one between-subjects factor (treatment) and one within-subjects factor (time). The statistical analysis was performed using IBM SPSS Statistics v26.0 (IBM, Armonk, NY, USA). All analyses were evaluated at the 0.05 level of significance.

## 3. Results

A total of 60 cats were enrolled in this part of the study. All cats recovered uneventfully postoperatively. Four cats in group G did not eat the gabapentin-containing creamy treat postoperatively and were excluded from the study. In addition, one group P cat had a postoperative pain score of 6 and was excluded from the study. Thus, a total of 55 cats completed this part of the study. The median (range) weight of cats was 3 kg (3–4 kg) in group G, 3.5 kg (3–5 kg) in group A, and 3 kg (3–4.5 kg) in group P. There was no difference in the median body weight among the three groups (*p* = 0.217, Kruskal-Wallis test). All postoperative values of biochemistry were within normal reference intervals and only mild abnormalities of CBC were noted, with the majority of cats presenting neutrophilic leukocytosis.

### 3.1. Stress Evaluation

Mean stress scores are presented in Table 1. There was no difference in the preoperative stress values among the three groups of cats. Eight hours postoperatively, the stress score was lower in group G (*p* < 0.01) and in group A (*p =* 0.01) compared to group P, but no difference was found between groups G and A (*p =* 0.59). Similarly, on the second postoperative day, the stress score was lower in group G (*p* < 0.01) and group A (*p* = 0.01) compared to group P, but no difference was found between groups G and A (*p* = 0.19).

Mean cortisol values are presented in Table 1. There was no difference in cortisol concentrations among the three groups at the time of their arrival in the clinic and at the end of the surgical procedure. On the second postoperative day, group G (*p* = 0.02) and group A (*p* = 0.01) had significantly lower serum cortisol compared to group P, but no difference was established between groups G and A (*p* = 0.78). The serum cortisol concentration immediately postoperatively was significantly less than the preoperative values in all groups (*p* < 0.01), but did not differ compared to the second postoperative day.

Mean values of glucose concentrations are presented in Table 1. After extubation, glucose was significantly lower in group A compared to groups P and G (*p* = 0.02 in both comparisons), but there was no difference between groups G and P (*p* = 0.94). On the second postoperative day, no difference was found among the three groups.

**Figure 2:**
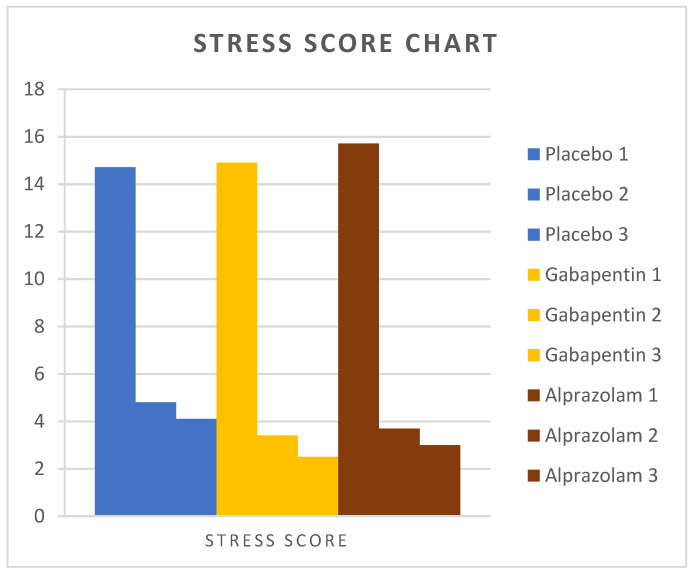
Stress scores were measured in cats from Groups P, G, and A. Assessments were conducted preoperatively, 8 h postoperatively, and 44 h postoperatively.

**Figure 3:**
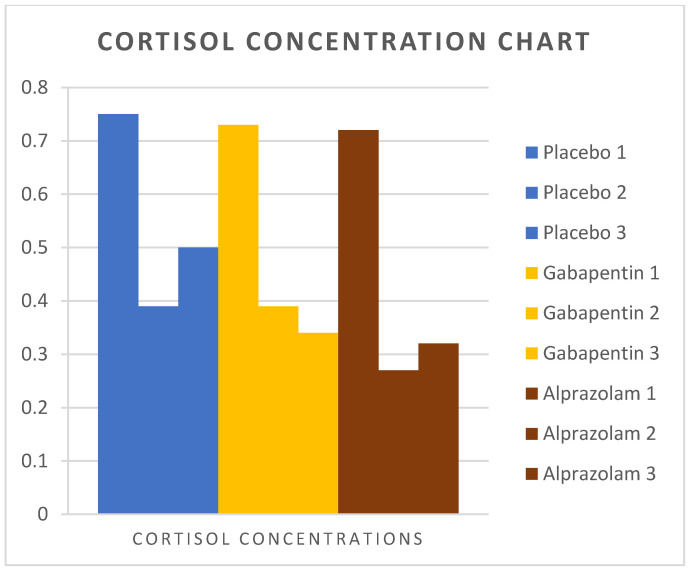
Cortisol concentrations were measured in cats from Groups P, G, and A. Measurements were taken preoperatively, immediately after surgery, and 44 h postoperatively.

**Figure 4:**
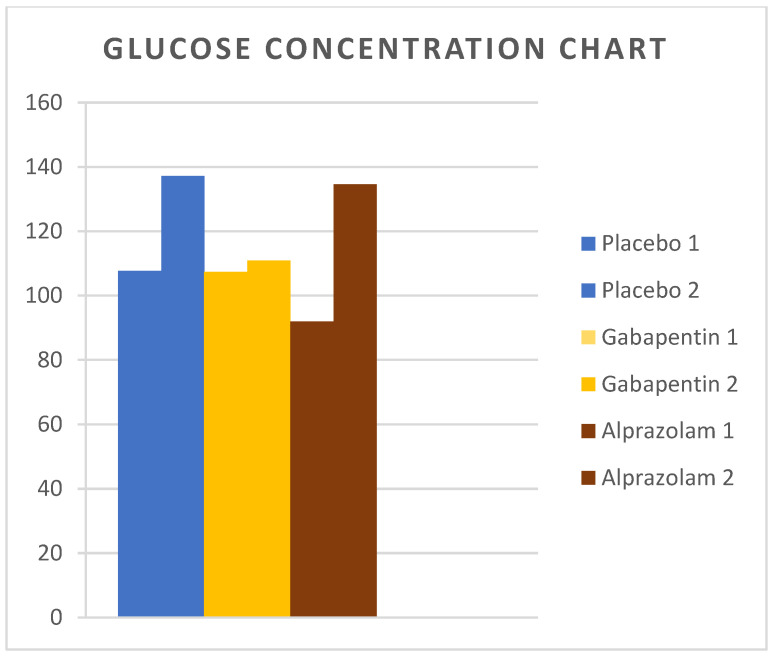
Glucose concentrations were measured in cats from Groups P, G, and A. Measurements were taken immediately after surgery and 44 h postoperatively.

Figure 2, Figure 3 and Figure 4 show the changes in measured stress variables over time in the three groups.

### 3.2. Food Consumption

**Figure 5:**
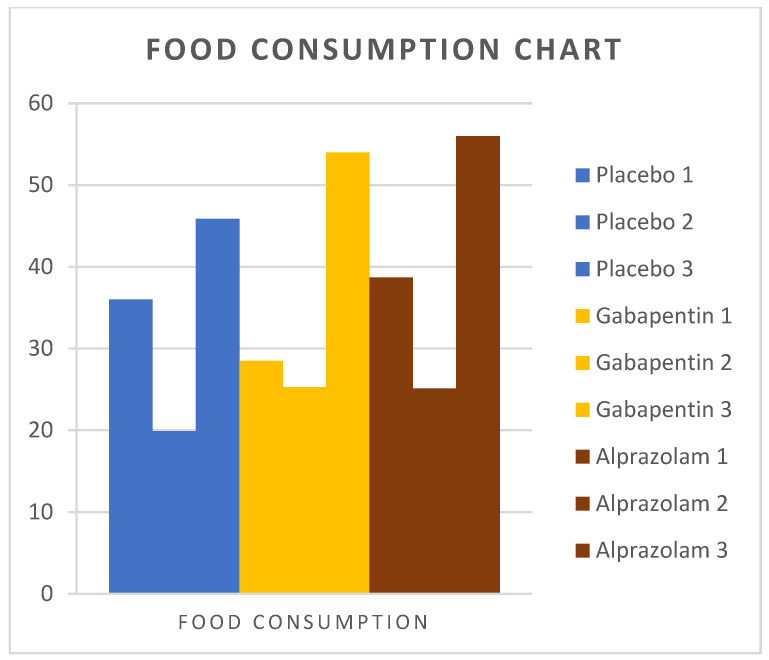
Food consumption was measured in cats from Groups P, G, and A. Measurements were taken on the first preoperative day of hospitalization, 8 h postoperatively, and on the first postoperative day.

The mean values of dry food consumption for the three time points are presented in Table 1. There was no difference among groups upon arrival and 8-h postoperatively. However, on the first postoperative day, food consumption was greater in group A compared to group P (*p* = 0.02), but no differences between groups P and G (*p* = 0.10) and between groups G and A (*p* = 0.44) were found. Changes in food consumption among groups over time are depicted in Figure 5.

### 3.3. Pain Assessment

**Figure 6:**
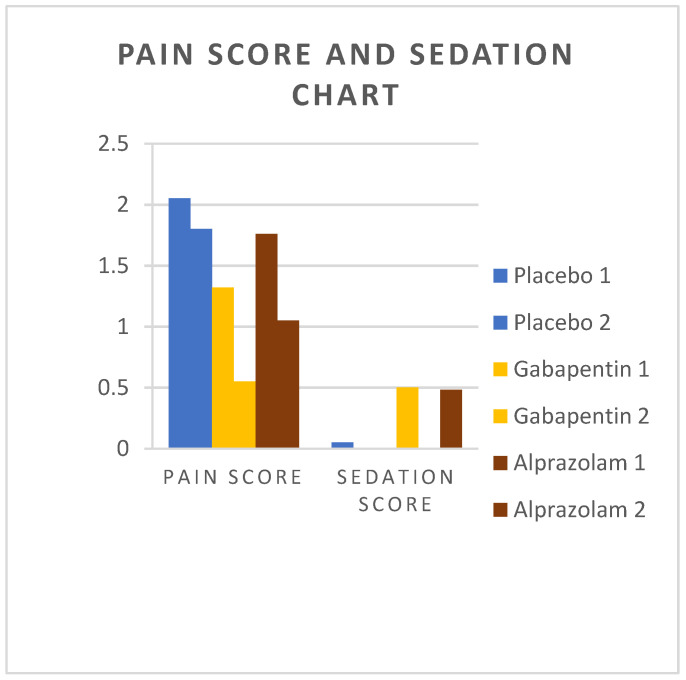
Pain scores and sedation scores were measured in cats from Groups P, G, and A. Measurements were taken 8 h postoperatively and on the second postoperative day.

Mean pain score values are presented in Table 1. There were no significant differences among groups 8 h postoperatively. However, the pain score of group G was significantly lower than that of group P on the second postoperative day (*p* = 0.01), but did not differ compared to that of group A (*p* = 0.33). Changes in pain scores of cats over time are presented in Figure 6.

### 3.4. Sedation Scores

Sedation was observed after treatment administration, with the majority of cats presenting ataxia. Mean values of sedation are presented in Table 1. There was no difference among groups 8 h postoperatively. On the second postoperative day, sedation was more severe in group A (*p* < 0.01) and group G (*p* < 0.01) compared to group P, but no difference was substantiated between groups G and A (*p* = 0.86). Changes in sedation scores among groups over time are presented in Figure 6.

## 4. Discussion

The results of the present study suggest that preoperative anxiolysis induced by gabapentin or alprazolam leads to a reduction in anxiety in cats after ovariohysterectomy. In addition, the administration of either gabapentin or alprazolam during short-term hospitalization after ovariohysterectomy significantly lowered stress levels and decreased serum cortisol of hospitalized cats compared to placebo-treated cats. On the other hand, gabapentin and alprazolam resulted in similar sedation levels postoperatively, which differed significantly compared to those in the placebo group.

Gabapentin has recently been used to address anxiety in cats before their veterinary appointment [23,24,25]. To the best of the authors’ knowledge, only one study has assessed the effects of long-term gabapentin administration in shelter cats, indicating an improvement in animals’ behavior and reduced stress levels after daily administration [16]. The results of our study align with the previous findings, indicating that daily gabapentin administration during short-term hospitalization substantially reduces the stress score in cats. This study provided evidence that the anxiolytic effect of alprazolam in cats during short-term hospitalization appears to be comparable to that of gabapentin. While alprazolam has been suggested to be beneficial in minimizing stress and fear responses in cats, no study has been published to demonstrate its efficacy [26]. In cats, alprazolam has been used for its anxiolytic properties as a component of the medical treatment of urethral obstruction [27,28]; however, its efficacy in reducing stress levels was not specifically evaluated. To the best of our knowledge, this study is the first to demonstrate the anxiolytic effects of alprazolam when administered during short-term hospitalization in cats. In addition, biochemical examination after the 2-day administration of alprazolam indicated that it can be administered safely in healthy cats, without any clinically relevant elevation in liver enzyme activity. Alprazolam is not metabolized by hepatic glucuronidation and is not expected to result in hepatic necrosis in cats [29]. Even though benzodiazepines may result in paradoxical aggression [15], no such effect was observed in our study.

Cortisol concentration can be increased by environmental stressors, which were kept to a minimum in our study [30], but it can also be increased postoperatively due to the activation of the hypothalamic–pituitary–adrenal axis (HPA) and the sympathetic nervous system [31]. In our study, gabapentin and alprazolam resulted in similarly decreased cortisol concentrations on the second postoperative day compared to placebo, possibly due to their anxiolytic effects. The impact of alprazolam on cortisol has also been documented in human studies, where significantly decreased cortisol concentrations were noted up to 2.5 h after alprazolam administration [32,33]. Regarding gabapentin, our findings are in contrast to the results of a previous study where gabapentin administration in cats did not affect serum cortisol concentration [30]; however, in our study, gabapentin was administered twice daily for two consecutive days, in contrast to the single dose administration of gabapentin in the previous study. The increased duration and frequency of gabapentin administration might account for the more noticeable change in cortisol concentrations. Importantly, in the latter study, cortisol was measured after dexmedetomidine was administered in all cats [30]. Dexmedetomidine is considered to hinder the effects of neurohumoral response and thus reduce the cortisol concentrations [34,35].

In the present study, serum cortisol upon admission was higher than the immediate postoperative values. This seemingly unexpected result is in agreement with the well-documented stress response in cats when first exposed to the veterinary clinic environment and medical procedures [36,37,38]. The impact of stressors in the hormonal response of cats is also emphasized in a previous study. In the latter study, cats whose front feet were bandaged had the same increase in serum cortisol concentrations as cats underwent onychectomy or tenectomy [36]. Results from our study also highlight the fact that the anticipated stress and anxiety of cats preoperatively can have a greater impact on the HPA axis and the sympathetic nervous system of cats than that induced from a surgical intervention.

Glucose evaluation is an indicator of stress in animals, as stressors provoke the release of catecholamines and cortisol, resulting immediately in stress-induced hyperglycemia. Furthermore, stressors can result in insulin resistance [31,39]. Cats in Group G failed to present significant difference in glucose concentrations compared to Group P. This result is in agreement with the findings of a previously published study [30]. In our study, alprazolam-treated cats had significantly lower glucose compared to other groups, suggesting its anxiolytic effect. Results of previous studies noted that alprazolam can attenuate the HPA axis and reduce the induced stress response [40], as well as increase insulin concentration when administered intraperitoneally in mice, non-significantly decreasing the glucose concentrations [41]. Glucose in group A did not differ from that in the other groups on the second postoperative day, despite the fact that stress scores and cortisol concentrations were more favorable in groups A and G compared to group P, likely indicating that the stress score and cortisol concentration might be more reliable proxy indicators of stress over time, compared to glucose values. Moreover, the significantly increased food intake in group A might partially account for the increase in glucose concentrations postoperatively.

In the present study, gabapentin was the only treatment that reduced postoperative pain scores of cats compared to the placebo-treated group, suggesting a potential analgesic effect, although superiority over alprazolam was not substantiated. Although human studies have shown that benzodiazepines given preoperatively can reduce postoperative pain through psychological factors [42], alprazolam is not considered to have analgesic effects postoperatively [43,44,45]. This observation is also reflected in the results of the present study, as postoperative pain scores of group A were similar to those of group P. Preoperative administration of gabapentin has been found to significantly decrease postoperative pain scores and the need of additional opioid analgesia in humans [46] and has also been shown to increase the time until patients warrant further analgesic medications [47]. In feline patients, gabapentin has been used successfully as an adjunct analgesic to treat chronic [48,49] and acute postoperative pain in those undergoing ovariohysterectomy [50].

Stress during hospitalization is a major cause of inappetence in cats [51]. Cats have a strong preference for a specific food texture [51]; thus, in the present study, only cats fed with dry food were eligible, to homogenize the feeding habits of the studied population. Published studies have indicated an increase in food intake after benzodiazepine administration in cats [26,52], as it is considered to have direct GABA-mediated central action in increasing food consumption [53]. These data agree with the results of this study, as food consumption in group A was significantly greater than that of group P on the first postoperative day. A mild, non-significant increase in food intake of group A compared to group P was also noted 8 h postoperatively. Gabapentin is also considered to have a positive impact on food consumption in cats postoperatively [54]. In our study, a mild but not significant increase in food intake was noted in group G compared to group P. The lack of a significant increase in food intake in our study might be the result of the different administration scheme compared to the previous study [54].

Both drugs used in this study induced sedation during hospitalization, primarily characterized by ataxia. It is known that both gabapentin and benzodiazepines have dose-related side effects like drowsiness, sedation, and ataxia [15,26,30]. Unlike previous findings, the current study indicates that alprazolam, 8 h postoperatively, did not result in increased sedation. One possible cause for the latter finding might be that, although the mean plasma elimination t1/2 after its oral administration is 11.2 h in human studies, there are no pharmacokinetic studies of alprazolam in veterinary medicine, so elimination time may be different in cats [15]. Similar to alprazolam, preoperative administration of gabapentin did not result in sedation 8 h postoperatively, but cats in group G showed increased sedation levels on the second postoperative day. The absence of sedation 8 h postoperatively may be attributed to the elimination half-life of gabapentin [55].

Several limitations are acknowledged in this study. A major limitation is that since there are no published studies on the pharmacokinetics of alprazolam in cats, the recommended doses and dosing intervals are currently extrapolated from human studies, which may have adversely affected the outcome of this study. Moreover, gabapentin powder was mixed with a treat and was not administered via its capsule. Pharmacokinetics of this method of administration have not yet been studied, even though other studies have also used similar techniques [54,56]. Finally, the efficacy of the tested treatments might be different in aggressive cats, which were excluded from this study. An additional potential limitation of the present study is that cortisol concentrations could have been influenced by the handling of the animals prior to the blood withdrawal. Restraining and the delay in time to obtain the blood can influence serum cortisol concentrations [57]. An alternative solution might be evaluating saliva cortisol concentrations; however, saliva samples cannot always be objectively evaluated [12].

## 5. Conclusions

In this study, both gabapentin and alprazolam demonstrated significant anxiolysis 8 h postoperatively and on the second postoperative day, as indicated by the Cat Stress Scores, serum cortisol concentrations, and, inconsistently, by glucose concentrations. While gabapentin led to reduced pain scores compared to placebo on the second postoperative day, only cats administered alprazolam exhibited a significant increase in food intake. Additionally, both treatments resulted in similar levels of sedation. Therefore, the findings of this study suggest that both gabapentin and alprazolam can be used during hospitalization of cats undergoing ovariohysterectomy. Further research is warranted to evaluate the efficacy of these treatments in different dosing regimens and in various animal species.

## Figures and Tables

**Table 1:** Variables assessed in 60 cats described as mean values ± SD for T1 (upon arrival), T2 (second preoperative day), T3 (after extubation), T4 (8 h postoperatively), T5 (first postoperative day), and T6 (44 h postoperatively).

Treatment		Stress Score	Cortisol Concentrations (ng L^−1^)	Glucose Concentrations (mg dL^−1^)	Food Consumption (g)	Pain Score	Sedation Score
Placebo	T1	14.7 ± 3.3	0.75 ± 0.57	NA	NA	NA	NA
	T2	NA	NA	NA	36 ± 17.6	NA	NA
	T3	NA	0.39 ± 0.23	107.7 ± 4.7 ^•^	NA	NA	NA
	T4	4.8 ± 1.9 *^†^	NA	NA	19.9 ± 11.1	2.05 ± 2.0	0.05 ± 0.2
	T5	NA	NA	NA	45.9 ± 15 ^□^	NA	NA
	Τ6	4.1 ± 1.6 ^‡■^	0.5 ± 0.22 ^‡■^	137.2 ± 10.7	NA	1.80 ± 2.2 ^‡^	0 ± 0 ^‡■^
Gabapentin	T1	14.9 ± 4.0	0.73 ± 0.42	NA	NA	NA	NA
	T2	NA	NA	NA	28.5 ± 21	NA	NA
	T3	NA	0.39 ± 0.26	107.4 ± 4.5 ^◊^	NA	NA	NA
	T4	3.4 ± 1.4 *	NA	NA	25.3 ± 8.3	1.32 ± 1.7	0 ± 0
	T5	NA	NA	NA	54 ± 11.7	NA	NA
	Τ6	2.5 ± 1.1 ^‡^	0.34 ± 0.25 ^‡^	110.9 ± 10.2	NA	0.55 ± 1.1 ^‡^	0.5 ± 0.5 ^‡^
Alprazolam	T1	15.7 ± 2.1	0.72 ± 0.26	NA	NA	NA	NA
	T2	NA	NA	NA	38.7 ± 21.7	NA	NA
	T3	NA	0.27 ± 0.16	91.9 ± 4.6 ^•◊^	NA	NA	NA
	T4	3.7 ± 0.9 ^†^	NA	NA	25.1 ± 8.4	1.76 ± 1.4	0 ± 0
	T5	NA	NA	NA	56 ± 7.6 ^□^	NA	NA
	Τ6	3 ± 1.2 ^■^	0.32 ± 0.19 ^■^	134.6 ± 10.5	NA	1.05 ± 1.5	0.48 ± 0.5 ^■^

* Statistically significant difference between groups P and G 8 h postoperatively. ^†^ Statistically significant difference between groups P and A 8 h postoperatively. ^‡^ Statistically significant difference between groups P and G 44 h postoperatively. ^■^ Statistically significant difference between groups P and A 44 h postoperatively. ^•^ Statistically significant difference between groups P and A after extubation. ^◊^ Statistically significant difference between groups G and A after extubation. ^□^ Statistically significant difference between groups P and A on the first postoperative day. NA—Not applicable.

## Data Availability

Data presented in this study are available on request from the corresponding author.
